# Supplementary material for: Conversion of Escherichia coli into Mixotrophic CO2 Assimilation with Malate and Hydrogen Based on Recombinant Expression of 2-Oxoglutarate:Ferredoxin Oxidoreductase Using Adaptive Laboratory Evolution
Source: Microorganisms. 2023 Jan 19;11(2):253. doi: 10.3390/microorganisms11020253 (PMC9967407; doi:10.3390/microorganisms11020253)
Supplement: Supplementary file 1 [file microorganisms-11-00253-s001.zip › 6. microorganisms-2014218-supplementary-final.pdf]

microorganisms

MDPI

Supplementary Information

1. Hydrogen dependence of microbial growth

We have done an exhaustive list of control experiment to show that hydrogen indeed serves as energy source. Figure S1 shows the growth with and without hydrogen for evolved, ancestral, and wild type with malate (25%) as carbon source and elevated CO_2(p_{CO2} = 0.2 atm). For wild type, Figure S2 shows the growth with and without hydrogen for ancestral with serine (100%) as carbon source at elevated CO_2 (p_{CO2} = 0.2 atm). For all conditions and strains, hydrogen either enhance or rescue the growth.

2. Discussion on possible nitrate usage as terminal electron acceptor

Although nitrate is added in our experiment as electron acceptor and *E. coli* can potentially metabolize malate or serine using nitrate as terminal electron acceptor in anaerobic aspiration [1-6], we observe no growth for wild type without H_2 in Figure S1C and Figure S2 and attempt to offer plausible quantitative explanation in terms of thermodynamics. Replacing oxygen (reduction potential+850mV)with nitrate (reduction potential +400mV) as terminal electron acceptor is thermodynamically unfavorable. For malate, it is possible to calculate maximal available Gibbs energy assuming that malate is converted to pyruvate by malate dehydrogenase and that the pyruvate is oxidized to acetylCoA via pyruvate dehydrogenase from complete oxidation in TCA cycle. In addition, we assume reducing power in NADH can be converted via oxidative phosphorylation in aerobic growth. Note that this value will represent upper bound of free energy available while neglecting energy expenditure for anabolism. Calculations were done using standard transformed Gibbs reaction energy data at pH= 7, ionic strength 0.25 M from the literature[7].

Malate dehydrogenase:

Malate + H_2O+ NAD_{ox} -> Pyruvate + NAD_{red} + $CO_{2(tot)}$

(Half reaction 1: -> Pyruvate + $CO_{2(tot)}$ + $2e^-$ -> Malate + H_2O $E_1'^0$ = -308 mV)

(Half reaction 2: NAD_{ox} +$2e^-$ -> NAD_{red} $E_2'^0$ = -316mV)

$\Delta G'° = nF(E_2'^0 - E_1'^0)$=-1.5 kJ/mol (n=2, F=96.7 $kJ.mol^{-1}V^{-1}$)

Pyruvate dehydrogenase:

Pyruvate + CoA + NAD_{ox} + H_2O -> $CO_{2(tot)}$ +NAD_{red} +aceyleCoA

$\Delta G'°$ = -30.48 kJ/mol

TCA cycle net reaction:

AcetylCoA + $3NAD_{ox}$ + FAD_{ox} +ADP + Pi + $4H_2O$ -> 2 $CO_{2(tot)}$ +3 NAD_{red} +FAD_{red} + ATP + CoA

$\Delta G'°$ = -53.2 kJ/mol

The net reaction is:

Malate + $6H_2O$+$5NAD_{ox}$ + FAD_{ox} +ADP + Pi -> 4 $CO_{2(tot)}$ +3 NAD_{red} + $5NAD_{red}$ +FAD_{red} + ATP + CoA

$\Delta G'°$ = -85.2 kJ/mol

We now convert Gibbs free energy and reducing power to ATP equivalent. First, energy is released in the form of free energy $\Delta G'°$ = -85.1 kJ/mol, or ~2.3 ATP equivalent, which is assumed to be reharvested. In terms of cofactor, reducing power is released in the form of 5 NAD_{red} (two electron carrier) and 1 FAD (two electron carrier) or equivalently 12 e^- is involved. In *E. coli*, when malate is oxidized aerobically, oxidative phosphorylation gives P/O ratio~1.25(the ratio of ATP formed per oxygen atom consumed) [8]. Four electrons are needed to oxidize oxygen molecule(two oxygen atoms) (with stoichiometry from half reaction O_2 +$4H^+$ +4 e^- -> H_2O). We can therefore convert reducing power to ATP equivalent (12/2)x1.25 = 7.5 ATP from reducing power. Adding these numbers including one ATP from TCA cycle, we obtain ~10.5 ATP per malate molecule.

We can calculate energy penalty with the standard reduction potential of oxygen and nitrate (BNID 104496) [7]. The reduction potentials represent the amount of energy that is released by donating an electron to one molecule. The oxygen/water pair has the greatest tendency to accept electrons. The nitrate/nitrite has lower reduction potential.

$O_2 + 4H^+ + 4e^- \rightarrow H_2O$ E'^0 (oxygen) = 850 mV

$NO_3^- + 2H^+ + 2e^- \rightarrow NO_2^- + H_2O$ E'^0 (nitrate) = 400 mV

Using the Nernst equation, $\Delta G'^\circ = -nFE'$(n=12 and F = 96.7 $kJ.mol^{-1}.V^{-1}$), the energy penalty when oxygen is replaced with nitrate as terminal electron acceptor is therefore $nF(E'^0$ (Oxygen/water)–E'^0 (nitrate/nitrite))= ~522 kJ/mol. This is equivalent to ~14 ATP using $\Delta G'^\circ$ ~-37 kJ/mol for ATP hydrolysis (BNID106580), which is much greater than ~10.5 ATP available when even malate is fully oxidized aerobically. We can see that the energy penalty is so huge that cell cannot extract positive net energy for growth from malate.

For serine, even aerobic growth of *E. coli* on serine as a carbon source is very poor, allowing doubling times of about ~60 h only [9]. Replacing oxygen with nitrate as terminal electron acceptor will make growth thermodynamically infeasible.

3. Disappearance of diauxic growth

The growth curves from 36th day and 40th day during malate evolution are displayed to show the disappearance of diauxic behavior in Figure S3. The growth curve from 36th day shows diauxic growth while the growth curve from 40th day shows single phase growth.

4. CO_2 dependence of ancestral and wild type strain

As control experiments, we also show the CO_2 dependence for ancestral and wild type strain (BW25113) with hydrogen supplied in Figure S4. In both cases, the growth under high CO_2 (p_{CO2} =0.2 atm) shows inferior growth rate as compared to N_2 purge and this is consistent with previous reports that CO_2 causes stress to the growth.

5. Calculation of growth rate and identification of diauxic growth

From the growth time-series data with ~ 10 min resolution, we use a non-parametric method to infer first derivatives as a function of time from time-series growth data[10]. We use the software download from http://swainlab.bio.ed.ac.uk/software/fitderiv.(accessed on 1 July 2020). The code is written in Python in Python 3 using NumPy, SciPy, Matplotlib, and the Pandas data analysis library. The code's capability to identify the diauxic shift of *S. cerevisiae* in a mixture of 0.4% glucose and 1% galactose was proven [10]. A representative growth curve with inferred growth rate at each time point is shown in Figure S5. The primary growth rate is 0.17 hr^{-1} at ~15 hr (first peak) and the secondary growth rate is 0.053 hr^{-1} at ~30 hr(second peak).

6. List of mutation points

We select isogentic clones from the 40th day of the first stage with serine and 144th day in second stage with malate evolution (designated as EVO-serine and EVO-malate) and sequence their genomes using Illumina whole genome sequencing. The complete list of SNPs is shown in Table S1.

7. Description of two other replicate evolution

In addition to the result presented in the main text, we have done two other replicate evolution. In short, samples are evolved for 56 days in serine for 1st stage and for 146 days in malate for 2nd stage. The evolved strain of these two evolution do not show mixotropic behavior. Moreover, in the 2nd stage of the evolution with malate, there is no diauxic growth behavior. We select isogentic clones from the 56th day of the first stage with serine and 146th day in second stage with malate evolution (designated as EVO-serine2 and EVO-

malate2 for 2nd evolution and EVO-serine 3 and EVO-malate3 for 3rd evolution) For reference, the complete list of SNPs is shown in Table S3 and Table S4. In general, the mutation result shows that in these two evolution the microbe sample seems to follow a very different route in the fitness landscape. Of note, there is no icd mutation found in these two replicate evolution.

8. Thermodynamic feasibility of OGOR reaction

The electron carrier used in OGOR reaction is ferredoxin. Unlike NADPH, ferredoxin (a one electron carrier) comes in various form with different midpoint reduction potential. For reference, well-studied Fe_4S_4 ferredoxin (from *Clostridium pasteruianum*), both have E'°= -420 mV [11]. More recently studied ferredoxins used in OGOR reaction from *Magnetococcus marinus* shows reduction potential ranging from E'°= -230 mV to E'°= -630 mV [12]. Ferredoxin from *E. coli* have reduction potential E'°= -380 mV [13]. We therefore would like to ask whether or not the ferredoxin from *E. coli* is thermodynamically potent enough to drive OGOR reaction. We resort to eQuilibrator—the biochemical thermodynamics calculator to directly calculate standard transformed Gibbs reaction energy [14]. To avoid ambiguity in different standard reduction potential of ferrexdoxin, we use standard transformed Gibbs reaction energy of the following reaction from eQuilibrator as the reference carboxylation reaction

$NADH_{red}$ + CO_2(aq) + Succinyl-CoA(aq) ⇌ NAD_{ox}+ CoA(aq) + 2-Oxoglutarate(aq)
$\Delta G'^{m}$ = 29 kJ/mol at PH= 7, pMg= 3, ionic strength 0.1 M

Half reaction 1: Succinyl-$CoA_{(aq)}$ + $CO_{2(tot)}$ + $2e^-$ -> $CoA_{(aq)}$ +2-Oxoglutarate(aq) $E_1'^0$
Half reaction 2: NAD_{ox} +$2e^-$ -> NAD_{red} $E_{NADH}'^0$ = -320 mV
$\Delta G_1'^{\circ} = nF(E_{NADH}'^0 - E_1'^0)$= 29 kJ/mol (n=2, F=96.7 kJ.mol^{-1}V^{-1})

We have omitted H^+ as a reactant as PH value is specified [7]. We wish to calculate the Gibbs reaction energy given NADH replaced with ferredoxin

$2Fd_{ox}$ + CO_2(aq) + Succinyl-CoA(aq) ⇌ $2Fd_{red}$+ CoA(aq) + 2-Oxoglutarate(aq)
Half reaction 1: Succinyl-$CoA_{(aq)}$ + $CO_{2(tot)}$ + $2e^-$ -> $CoA_{(aq)}$ +2-Oxoglutarate(aq) $E_1'^0$
Half reaction 2: $2Fd_{ox}$ +$2e^-$ -> Fd_{red} $E_{FD}'^0$ = -380 mV
$\Delta G_2'^{\circ} = nF(E_{FD}'^0 - E_1'^0) = nF\ E_{FD}'^0 + \Delta G_1'^{\circ} - nF\ E_{NADH}'^0$ =17.4 kJ/mol
(n=2, F=96.7 kJ.mol^{-1}V^{-1})

The standard reduction potential of NADH is $E'_{NADH}{}^{\circ}$= -320 mV [13]and the Gibbs free energy difference when NADH is replaced with ferredoxin $E'_{FD}{}^{\circ}$ =-380mV is hence $nF(E'_{FD}{}^{\circ} - E'_{NADH}{}^{\circ})$ where n and F are number of electrons and Faraday constant (n= 2 and F = 96.7 kJ.mol^{-1}V^{-1}). The reducing power of ferredoxin of *E. coli* is more potent than that of NADH. Hence the standard transformed Gibbs reaction energy will be lower. For *E. coli*, the computed value is $\Delta G_2'^{\circ}$ ~17.4 kJ/mol, satisfying the reversibility criteria i.e., $\Delta G'^{\circ}$ < 30 kJ/mol [15]. Indeed, the result is consistent with the enzyme activity data from *Magnetococcus marinus* [12]. The OGOR reaction was proven feasible *kinetically* using one ferredoxin $MmFd_2$ (from *Magnetococcus marinus*) with reduction potential E'°=-380mV.

9. Strain and genomic modification

The E. coli was derivative from BW25113 strain containing a plasmid pGETS-KorAB (Cm^r). Then a plasmid pCC1Gm-pR-evo-icd (Gm^r, was not sequenced) containing mutated icd gene with 11 mutation points from Table S1 was transformed into this strain. Therefore, this *E. coli* strain was BW25113 with plasmids pGETS-KorAB and pCC1Gm-pR-evo-icd. The genotype can display like: *Δ(araD-araB)*567 *ΔlacZ4787*(::rrnB-3) *λ- rph-1 Δ(rhaD-rhaB)568 hsdR514* / pGETS-118 Cm^r *korAB*, pCC1-4k Cm^r::Gm^r evo-*icd*

Data availability: To make the experimental data avaialble, we have submitted the NGS data to ncbi database and the link to the data is https://www.ncbi.nlm.nih.gov/sra/PRJNA782208. (accessed on 01 August 2021)

References

1. Boonstra, J. and Konings, W.N. Generation of an electrochemical proton gradient by nitrate respiration in membrane vesicles from anaerobically grown *Escherichia coli.* Eur. J. Biochem. **1977**, 78, 361-368.
2. Garland, P.B., Downie, J.A. and Haddock, B.A. Proton translocation and the respiratory nitrate reductase of *Escherichia coli*. Biochem. J. **1975**, 152, 547-559.
3. Blasco, F., Iobbi, C., Ratouchniak, J., Bonnefoy, V. and Chippaux, M. Nitrate reductase of *Escherichia coli* : sequence of the second nitrate reductase and comparison with that encoded by the narGHJI operon. Mol. Gen. Genet. **1990**, 222, 104-111.
4. Blasco, F., Nunzi, F., Pommier, J., Brasseur, R., Chippaux, M. and Giordano, G. Involvement of the narJ or narW gene product in formation of active nitrate reductase in *Escherichia coli*. Mol. Microbiol. **1992**, 6, 209-219.
5. Grove, J., Tanapongpipat, S., Thomas, G., Gri.ths, L., Crooke, H. and Cole, J. *Escherichia coli* K-12 genes essential for the synthesis of c-type cytochromes and a third nitrate reductase located in the periplasm. Mol. Microbiol. **1992**, 19, 467-481.
6. Darwin, A.J., Ziegelho¡er, E.C., Kiley, P.J. and Stewart, V. Fnr, NarP and NarL regulation of *Escherichia coli* K-12 napF (periplasmic nitrate reductase) operon transcription in vitro. J. Bacteriol. **1998**, 180, 4192-4198.
7. Alberty, R. A. Thermodynamics of Biochemical Reactions. **2003** John Wiley & Sons, Inc.
8. Orth J, Fleming R, Palsson B. (2012) Reconstruction and use of microbial metabolic networks: the core *Escherichia coli* metabolic model as an educational guide. EcoSal Plus 2010. https://doi.org/10.1128/ecosalplus.10.2.1.
9. Zinser, E. R., and Kolter. R. Mutations enhancing amino acid catabolism confer a growth advantage in stationary phase. J. Bacteriol. **1999**, 181:5800-5807.
10. Swain, P. S. et al. Inferring time-derivatives, including cell growth rates, using Gaussian processes. Nat. Commun. **2016**, 7:13766.
11. Prince, R.C. and Adams, M. W. Oxidation-reduction properties of the two Fe_4S_4 clusters in *Clostridium pasteurianum* ferredoxin, J. Biol. Chem. **1987**, 262 5125–5128.
12. Chen, P.Y.T.; Li, B.; C. L. Drennan, C.L.; Elliot S.J. A reverse TCA cycle 2-oxoacid;ferredoxin oxidoreductase that makes C-C bonds from CO2. Joule. **2019**, 3, 595-611.
13. Knoell H.E.; J. Kappe J. *Escherichia coli* ferredoxin, an iron-sulfur protein of the adrenodoxin type. Eur. J. Biochem. **1974**, 50 245-52.
14. Flamholtz, A., Noor, E., Bar-Even, A. and Milo, R. eQuIlibrator — the biochemical thermodynamic calculator. *Nucleic Acid Research . Syst.* **2011**, 1-6.
15. Bar-Even A, Flamholz A, Noor E, and Milo R. Thermodynamic constraints shape the structure of carbon fixation pathways. Biochim Biophys Acta. **2012**, 1817:1646–59.

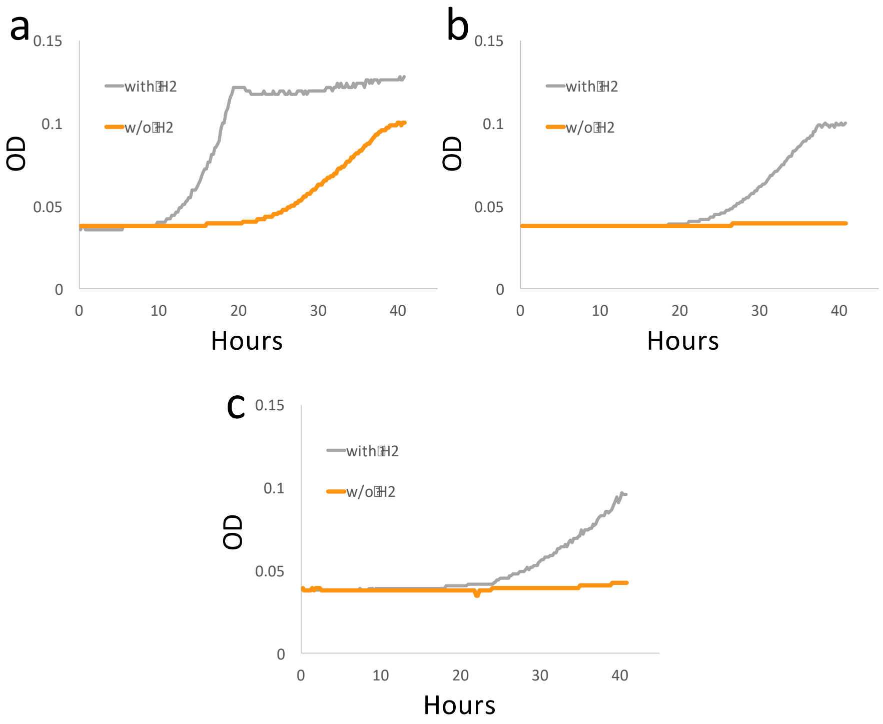

Figure S1. Hydrogen dependence of growth for the evolved, ancestral, and wild type strain with malate as carbon source**.** (**a**) evolved clone. (**b**) ancestral strain (**c**) wild type strain. For all data, growth is done with 25% malate concentration and elevated CO_2(p_{CO_2} = 0.2 atm).

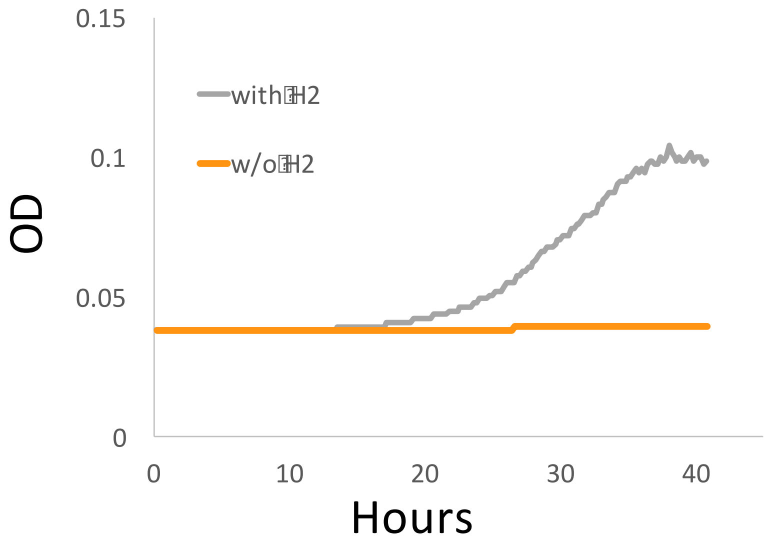

Figure S2. Hydrogen dependence of growth for ancestral strain with serine as carbon source.

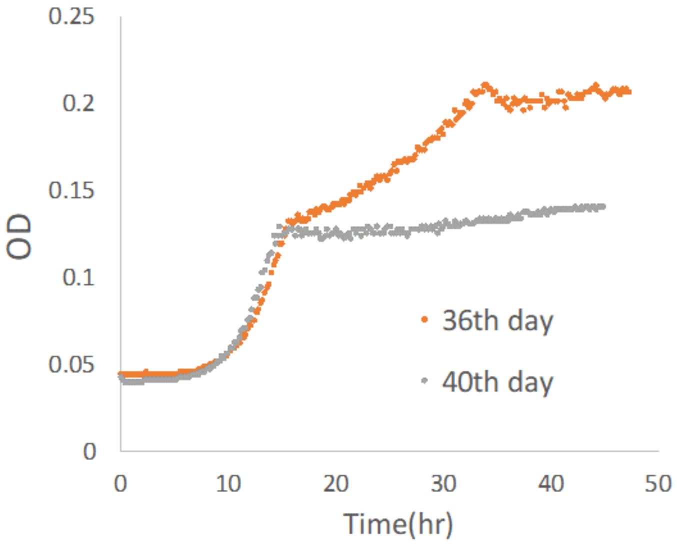

Figure S3. Representative growth curve before and after disappearance of diauxic growth with 25% malate concentration and hydrogen supply. Growth curve from 36th day sample shows diauxic growth while growth curve from 40th day shows single phase growth.

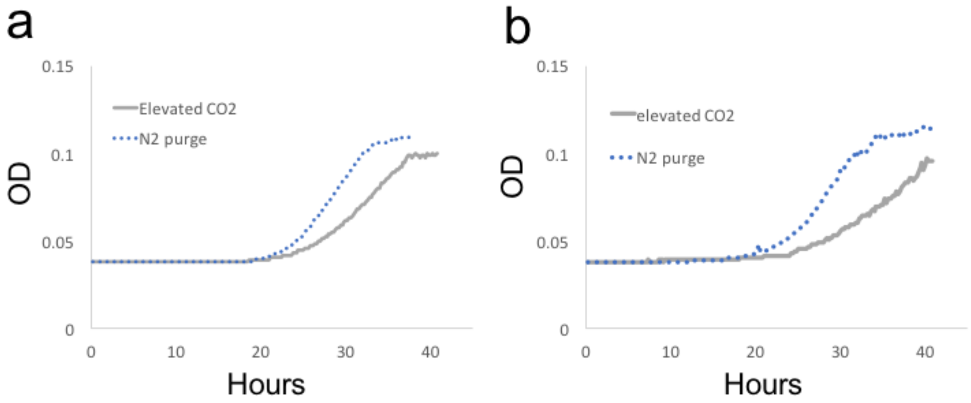

Figure S4. CO_2 dependence of growth for ancestral and wild type strain with hydrogen supplied. (**a**) Ancestral strain. The growth rates for N_2 purge and elevated CO_2 are 0.113 ± 0.017 hr^{-1} and 0.0572 ± 0.0013 hr^{-1}, respectively. (**b**) Wild type strain. The growth rates for N_2 purge and elevated CO_2 are 0.105 ± 0.04 hr^{-1} and 0.073 ± 0.006 hr^{-1}, respectively.

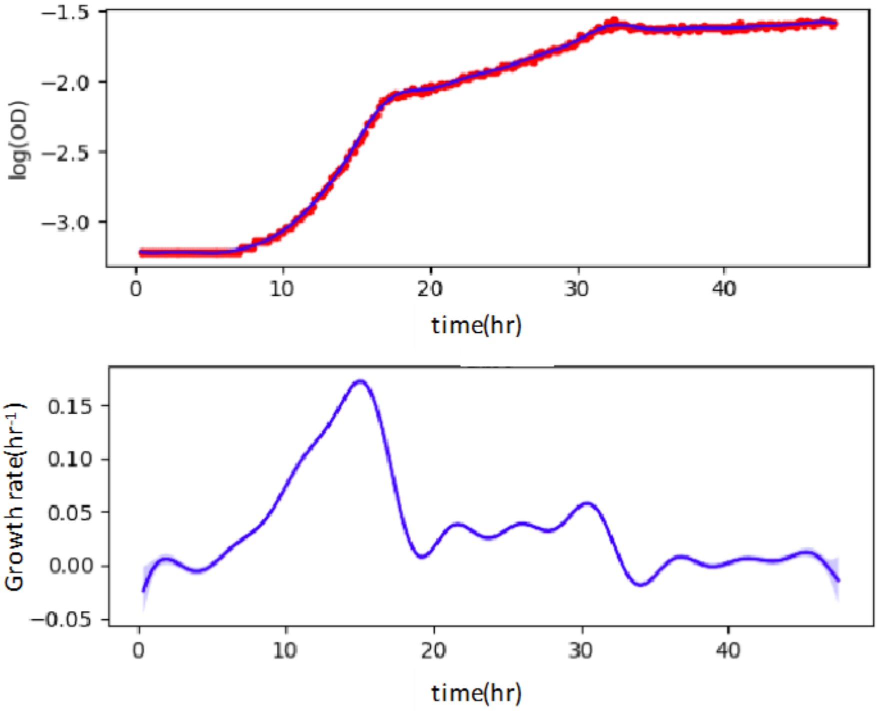

Figure S5. Representative growth rate calculation. A growth curve on 34th day during the second stage evolution with malate showing a diauxic shift. The best-fit function is shown in dark blue and the inferred growth rate is shown below. The primary growth rate is 0.17 hr^{-1} at ~15 hr (first peak) and the secondary growth rate is 0.053hr^{-1} at ~30 hr(second peak).

Table S2. Differential gene expression of *E. coli* evolved and ancestral strains.

Gene name	Description	Evolved strain (TPM)[a]	Ancestral strain (TPM)	Log2ratio (Evolved /Ancestral)[b]
**Heterologous expression (*Chlorobaculum tepidum* genes cloned into *Escherichia coli*)**				
korA (CT0163)	α-oxoglutarate ferredoxin oxidoreductase subunit α	432,562	424,176	0.028
korB (CT0162)	2-oxoglutarate ferredoxin oxidoreductase subunit β	26,719	3158	3.080
***E. coli* genes**				
**Galactitol degradation**				
gatD	galactitol-1-phosphate dehydrogenase, Zn-dependent and NAD(P)-binding	3.4	4549.4	-10.357
gatC	galactitol PTS permease - GatC subunit	4.1	6124.8	-10.524
gatB	galactitol-specific enzyme IIB component of PTS	4.2	9966.1	-11.201
gatA	galactitol-specific enzyme IIA component of PTS	2.9	7433.4	-11.291
gatZ	D-tagatose 1,6-bisphosphate aldolase 2, subunit	2.5	15,246.5	-12.573
gatY	D-tagatose 1,6-bisphosphate aldolase 2, catalytic subunit	0.1	19,305.7	-16.751
**Gluconeogenesis**				
fbaB	fructose-bisphosphate aldolase class I	0.0001	111.2	-20.085
**Tryptophan:proton symport**				
mtr	tryptophan transporter of high affinity	1185.7	3.0	8.592

[a]TPM indicates transcripts per million.
[b]Differential gene expression was analysed with Student's *t*-test and the false discovery rate, and both *p*-values and *q*-values < 0.05 are shown, except heterologous genes on plasmid.
